# Expression and function of cartilage-derived pluripotent cells in joint development and repair

**DOI:** 10.1186/s13287-020-01604-y

**Published:** 2020-03-12

**Authors:** Zhou Jiang, Sijing Yu, Hengyi Lin, Ruiye Bi

**Affiliations:** 1 State Key Laboratory of Oral Diseases, National Clinical Research Center for Oral Diseases, West China Hospital of Stomatology, Sichuan University, Chengdu, China; 2 Department of Orthognathic and TMJ Surgery, State Key Laboratory of Oral Diseases, National Clinical Research Center for Oral Diseases, West China Hospital of Stomatology, Sichuan University, Chengdu, China

**Keywords:** Cartilage-derived pluripotent cell, Chondroprogenitor cell, Stem cell-based therapy, Cartilage repair, Stem cell transplantation

## Abstract

Cartilage-derived pluripotent cells reside in hyaline cartilage and fibrocartilage. These cells have the potential for multidirectional differentiation; can undergo adipogenesis, osteogenesis, and chondrogenesis; and have been classified as mesenchymal stem cells (MSCs) conforming to the minimal criteria of the International Society for Cellular Therapy. Cartilage tissue is prone to injury and is difficult to repair. As cartilage-derived pluripotent cells are the closest cell source to cartilage tissue, they are expected to have the strongest ability to differentiate into cartilage compared to other MSCs. This review focuses on the organizational distribution, expression, and function of cartilage-derived pluripotent cells in joint development and repair to help explore the therapeutic potential of in situ cartilage-derived pluripotent cells for joint cartilage repair.

## Background

Articular cartilage in long bones is made up of hyaline cartilage. The condylar cartilage (CC) located in the temporomandibular joint (TMJ) is generally considered fibrocartilage. The articular disc, including the meniscus and the TMJ disc, is also composed of fibrocartilage. Due to the lack of nerves, blood vessels, and lymphatic vessels and the effect of its weight-bearing role, cartilage tissue shows difficulty repairing itself when injured.

With the rise of regenerative medicine and tissue engineering, cell-based approaches have been successfully used in cartilage repair. Both autologous chondrocytes and mesenchymal stem cells (MSCs) are currently used as seed cells for repairing cartilage injury. However, the amount of healthy cartilage available for chondrocyte harvesting is often limited during autologous chondrocyte transplantation. Chondrocyte phenotypes are difficult to maintain during culture expansion, and these cells are prone to dedifferentiating and losing their capacity to form cartilage. Instead, MSCs are considered a preferable cell source for cartilage repair because they are easy to isolate, retain some stem cell properties during in vitro expansion, and can differentiate into chondrocytes.

MSCs can be isolated from the bone marrow [1], periosteum [2], synovium [3], and adipose tissue [4]. Generally, the closer the cell source is to the injured cartilage tissue, the more effective the differentiation into cartilage tissue is [5]. Therefore, if MSCs are also present in the articular surface, they are expected to have the strongest ability to differentiate into cartilage and repair injured cartilage tissue.

Recent studies have found that articular cartilage contains pluripotent cell populations that can undergo chondrogenic, osteogenic, and adipogenic differentiation. These cells have been classified as MSCs conforming to the minimal criteria of the International Society for Cellular Therapy, which include being plastic-adherent, showing multipotentiality, and expressing an MSC marker phenotype [6, 7]. Therefore, these populations are expected to be potential cell sources for cartilage repair, and in-depth and comprehensive studies on their function in joint development and repair can help us explore ideal stem cell-based therapies for cartilage repair. Since these cells had various names in different studies, we named these cells cartilage-derived pluripotent cells in our study.

## Organizational distribution of cartilage-derived pluripotent cells

### In long bones

#### In hyaline cartilage

**Fig. 1:**
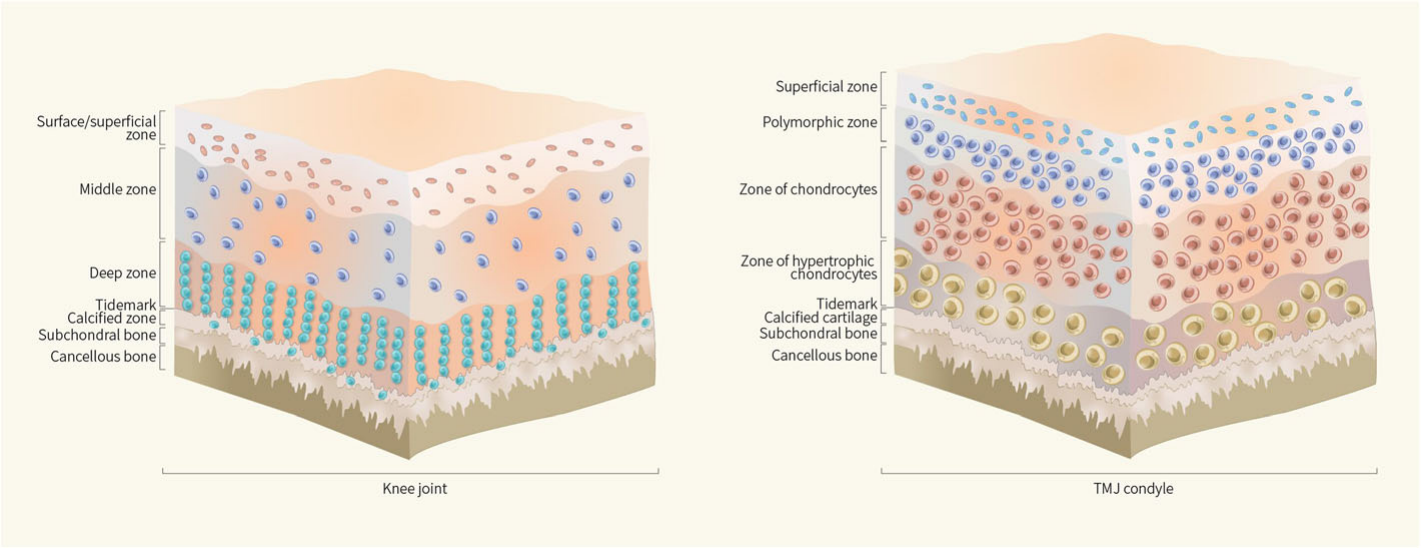
Zonal structure of cartilage. **a** Hyaline cartilage is compartmentalized into the surface zone, middle zone, deep zone, and calcified zone. **b** Fibrocartilage in TMJ condyle is divided into four distinct zones: the fibrous SZ, a polymorphic zone, a zone of chondrocytes, and a zone of hypertrophic chondrocytes

Hyaline cartilage is compartmentalized into the surface zone, middle zone, deep zone, and calcified zone (Fig. 1a), with biochemical and morphological variations existing at different depths [8]. Multiple studies have confirmed the presence of pluripotent cells with stem cell characteristics in hyaline cartilage [6, 9, 10], and the surface zone of the cartilage tissue, including the articular surface, is a relatively abundant source of these pluripotent cells. In the development of articular cartilage, Hayes et al. [11] found that articular surface zone cells from animal knee joints had a longer cell cycle than the underlying transitional zone cells, and Hunziker et al. [12] found that the superficial zone (SZ) consisted of slowly dividing stem cells, which suggested the presence of a chondroprogenitor or stem cell population in the articular cartilage surface. Further, Dowthwaite et al. [8] and Hattori et al. [9] both successfully isolated stem/progenitor cells from the surface zone of calf/bovine articular cartilage, and the latter study reported that these progenitors make up approximately 0.1% of all cells that can be extracted from the surface zone of the articular cartilage tissue. Grogan et al. [13] found that the frequency of progenitor cells in full-thickness human articular cartilage was 0.14%, and no difference was found between the control and osteoarthritis (OA) groups. Interestingly, Pretzel et al.’s [14] study indicated a much higher percentage of CD105+/CD166+ progenitors in OA (16.7%) cartilage compared to normal (15.3%) cartilage, and the CD166+ cells were almost exclusively located in the superficial and middle cartilage zones. A recent study demonstrated that high-efficiency colony-forming cells (HCCs) can also be isolated from the deep zone of bovine articular cartilage, although the SZ has significantly more progenitor cells than the deep zone [15].

#### In meniscus

A recent study showed that multipotent stem cells are present in the human meniscus and are phenotypically similar to MSCs [16]. Shen et al. [17] identified and characterized a population of meniscus-derived stem cells (MeSCs) that displayed low immunogenicity and possessed immunosuppressive functions. Then, this team found that human meniscus stem/progenitor cells (hMeSPCs) displayed both MSC characteristics and high expression levels of type II collagen [18]. Similarly, another study showed that MSCs isolated from rabbit menisci have universal stem cell characteristics, including clonogenicity, multipotency, self-renewal capacity, and expression of stem cell markers, and a pronounced tendency to chondrogenic differentiation appeared both in vivo and in vitro compared to that of bone marrow-derived stem cells (BMSCs) [19]. Gamer et al. [20] isolated and localized stem/progenitor cells from murine menisci grown in explant culture, and localization studies suggested that endogenous progenitor cells may reside in the superficial and outer regions of the meniscus in vivo.

### In TMJ fibrocartilage

**Fig. 2:**
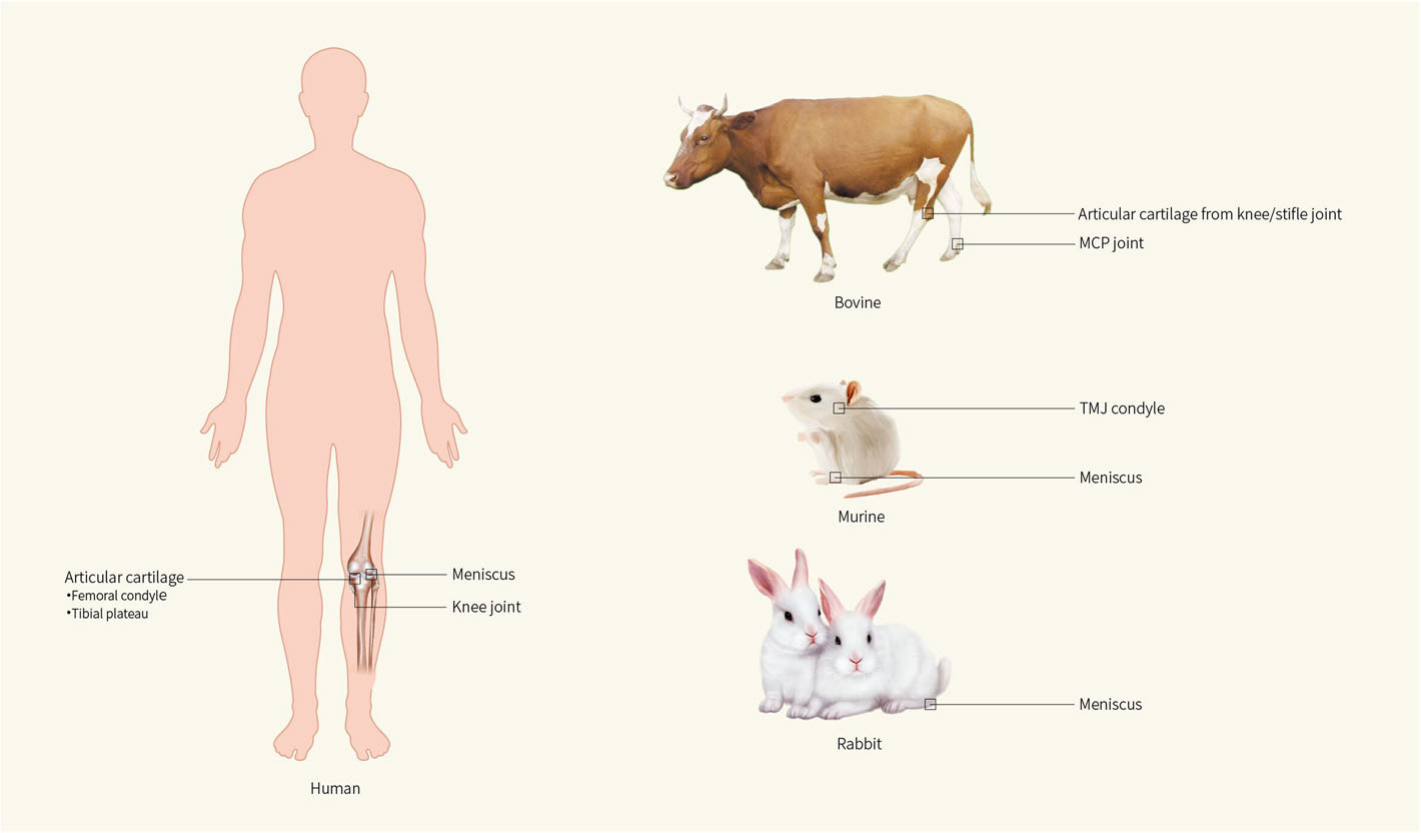
Organizational distribution of cartilage-derived pluripotent cells. Cartilage-derived pluripotent cells are mainly isolated from human, bovine, murine, and rabbit, where these pluripotent cells are located in each species is shown

Unlike hyaline cartilage, fibrocartilage in the TMJ condyle consists of various proportions of both fibrous and cartilaginous tissue and is divided into four distinct zones (Fig. 1b): the fibrous SZ, a polymorphic zone, a zone of chondrocytes, and a zone of hypertrophic chondrocytes [21]. In the fibrocartilage of the TMJ, Embree et al. [22] first showed that the fibrous SZ tissue in the TMJ condyle is a niche that harbors fibrocartilage stem cells (FCSCs). The skeletal stem/progenitor cell marker αSMA was traced in transgenic mice [23] by pedigree tracking studies, and the researchers found that the mature col2a1+ chondrocyte progeny located in CC tissues were differentiated from undifferentiated αSMA+ cells in the fibrous SZ, indicating that the fibrocartilage stem cells in the SZ can produce mature chondrocytes (Fig. 2).

## Cytological features of cartilage-derived pluripotent cells

Although cartilage-derived pluripotent cells currently lack definitive biomarkers, these cells are generally characterized according to several cytological features, such as increased chondrogenic protein expression, specific cell surface markers, a high colony-forming efficiency, a pluripotent differentiation capacity, and migratory ability.

### Increased chondrogenic protein expression during cartilage development

Cartilage development is a continuous dynamic process of cell differentiation and protein expression. In hyaline cartilage, pluripotent cells at different stages of differentiation exhibit expression of different genes, including fibronectin, hyaluronan, tenascin, type I collagen, type II collagen, minor type IX and XI collagens, proteoglycans, matrilins, and cartilage oligomeric protein (COMP) [24–27], which participate in the formation of abundant extracellular matrix (ECM). In this process, undifferentiated mesenchymal cells eventually differentiate into chondrocytes. In mature articular cartilage, the cells of the surface zone produce high quantities of SZ protein (SZP/proteoglycan 4), encoding lubricin for surface lubrication [28, 29]. In the middle and deep zones, the cartilage ECM is composed mainly of type II collagen, aggrecan, and hyaluronan [30]. Assays of these chondrogenic proteins and their corresponding mRNA/gene levels, especially those of type II collagen, aggrecan, and COMP, can be used to measure the chondrogenic differentiation potential of specific cell populations.

### Surface markers

Cartilage-derived pluripotent cells are generally characterized according to MSC-related surface markers, which can effectively distinguish them from chondrocytes. These pluripotent cells express the classical MSC markers CD105, CD73, and CD90 and lack expression of CD45, CD34, CD14 or CD11b, CD79a, or CD19, according to the International Society for Cellular Therapy [7]. CD10 and CD166 (ALCAM) are also regarded as distinctive markers for MSCs [31, 32]. In addition, these pluripotent cells express stem cell markers, such as Stro-1, Notch1, VCAM-1 (CD106), and Integrin β1 (CD29) [8, 10, 13, 33], and chondrogenic markers, such as Sox9 [6, 10]. These surface markers were also expressed in combinations, including CD9^+^/CD90^+^/CD166^+^ for OA [34], CD166^+^/CD90^+^ for normal [35], and CD105^+^/CD166^+^ for both normal and OA samples from human articular cartilage [14].

Recent studies have shown that cartilage-derived pluripotent cells also express specific surface markers. Grogan et al.’s [36] study found that in normal human knee joints, chondrocytes with a high chondrogenic capacity expressed increased levels of CD44, CD49c, CD49f, and CD151. CD90 and CD166 were also highly expressed, which suggests that these highly chondrogenic subpopulations might correspond to those with progenitor characteristics. Williams et al. [6] found that CD49e might be utilized as a specific marker for the cartilage progenitor cell population in normal human articular cartilage from femoral condyles. In knee joints from late-stage OA, CD146 might be a new cell surface marker for the cartilage progenitor cell population [37]. Another study compared protein changes between human bone marrow MSCs and chondrogenic progenitor cells (CPCs) from knee articular cartilage from OA samples, and 4 cell surface proteins were found with significantly increased expression in the CPCs: AMPN (CD13), CD109 antigen, CADM1, and CD49b [38].

However, as these studies examined cartilage-derived pluripotent cells from different species and with a different cell origin and cartilage status (normal or OA), the surface markers currently used for identification are not unified. Therefore, the identification of specific markers to effectively distinguish cells that can differentiate into cartilage is needed.

### Colony formation

Colony-forming ability is a well-recognized trait of stem/progenitor cells that has been used extensively to perform quantitative and functional analysis of clonal populations of progenitors [39], and cartilage-derived pluripotent cells have a strong clonogenic potency.

In hyaline cartilage, Dowthwaite et al. [8] defined a colony as more than four cells and found that articular surface cells with high affinity for fibronectin showed a significantly enhanced colony-forming efficiency (CFE) relative to all other cohorts. Compared to nonclonal dedifferentiated chondrocytes, immature bovine chondroprogenitor cells from the metacarpophalangeal (MCP) joints showed 2.6-fold greater telomerase activity and significantly longer telomere lengths of chromosomes during long-term clonal expansion in a monolayer culture [40]. Williams et al. [6] found that clonal cartilage progenitor cells isolated from human articular cartilage could proliferate to over 60 population doublings (PD) cultured in monolayers, taking over 200 days. Similarly, in the TMJ condyle, FCSCs formed sixfold more colonies than CC cells [22].

Interestingly, CPCs from articular cartilage of the later stages of human OA were also found to undergo 60 PD in monolayer culture, with incrementally increasing time for each doubling event [10]. Another study showed that articular cartilage-derived CPCs from cartilage from OA samples can be subdivided into two populations: an early senescent population (ES-OA-CPCs) that underwent replicative exhaustion by 30 PD and a late senescent population (LS-OA-CPCs) that was capable of prolonged expansion and displayed similar growth rates compared to stem cells from normal cartilage [39]. These findings suggested that although early senescence is an inherent property of a subset of activated progenitors, there is also a pool of progenitors with extended viability and regenerative potential residing within cartilage from OA samples.

### Pluripotent differentiation

The potential for chondrogenic, osteogenic, and adipogenic differentiation has become a defining feature of cartilage-derived pluripotent cells that distinguishes them from mature chondrocytes. Progenitors from the articular cartilage surface treated with bone morphogenic protein-7 (BMP-7) showed robust chondrogenesis and produced ECM for cartilage [9]. Studies have also found that CPCs from both normal articular cartilage and OA-derived stem cell populations demonstrate trilineage differentiation into adipogenic, osteogenic, and chondrogenic [6, 41] lineages, suggesting that stem cells from human OA cartilage also have the potential for cartilage repair. Koelling et al. [10] observed that CPCs from knee joints from late-stage OA regained a round chondrocyte-like phenotype and exhibited collagen type II mRNA expression as well as collagen type II protein expression 3 weeks after transfer to a 3D-alginate culture without any chondrogenic supplementation. High mRNA levels of sox-9 and collagen type II and low levels of runx-2 and collagen type I were also identified in these cells. Moreover, CPCs adhere to and are influenced by ECM components, and downregulation of runx-2 enhances their chondrogenic potential. Subsequently, another study showed that CD146+ chondroprogenitors from knee joints of late-stage OA showed a lower potential for adipogenesis and osteogenesis but a much higher potential for chondrogenesis compared to unsorted chondrocytes and adipose-derived MSCs, as these CD146+ cell subpopulations showed increased type II collagen, aggrecan, and Sox9 expression [37]. In TMJ condyles, FCSCs underwent adipogenesis, chondrogenesis, and osteogenesis, and their individual colonies showed heterogeneous differentiation potential (22.5% trilineage, 64.5% bilineage, and 12.9% single lineage) [22].

### Migratory ability

Migratory ability enables cartilage-derived pluripotent cells to migrate to the injured site and repair cartilage damage. Koellings et al. [10] detected CPCs in degenerated cartilage sites in late-stage OA and found that CPCs not only migrated in vitro but also populated diseased tissue ex vivo. Seol et al. [42] observed that CPCs migrated to the site of injury caused by blunt impact or scratching in healthy cartilage explants from mature cattle, and these migrating cells were highly clonogenic and multipotent and expressed CPC-associated markers, including CD105, CD73, CD90, CD29, CD44, Notch-1, and Sox9. The latter study also suggested that migratory cartilage-derived pluripotent cells might exist in healthy cartilage.

## In situ cartilage-derived pluripotent cell-based therapies for joint cartilage repair

Articular cartilage is a type of tissue that is easily damaged and difficult to repair. Currently, MSC-based therapies, mainly BMSCs, are widely used to effectively repair cartilage defects [43]. In recent years, researchers have discovered a special population of stem cells, in situ cartilage-derived pluripotent cells, which reside in both healthy and injured joint cartilage/fibrocartilage tissues and exhibit strong repair capabilities. There are two main strategies for in situ cartilage-derived pluripotent cell-based therapies for joint cartilage repair. One is to transplant cartilage-derived pluripotent cell-containing grafts into the cartilage defect, and the other is the intra-articular injection of pluripotent cells (Table 1). Nevertheless, in situ cartilage-derived pluripotent cells display different reparative characteristics in hyaline cartilage, meniscus, and TMJ condyle.

**Table 1:** Cartilage-derived pluripotent cell-based therapies for joint cartilage repair

Cartilage	Cell	Source	Species	Model	Mode of action	Study/evaluation	In vivo/in vitro	Result	Conclusion	Year	Author
Hyaline cartilage	Autologous chondroprogenitor cells	Articular cartilage	Equine	15 mm cartilage defects on the medial trochlear ridge of the femur	A graft: autologous chondroprogenitor cells transplanted in a fibrin matrix	Lameness (pain), arthroscopic, radiographic, gross, histologic, and immunohistochemical analyses	In vivo	Improved the amount of type II collagen and decreased central osteophyte formation	Had significantly better repair tissue	2015	Frisbie et al. [44]
	Chondroprogenitors	Articular cartilage	Goat	A circular 6 mm defect in the lateral femoral condyle	A graft: Chondro-Gide® membrane seeded with goat chondroprogenitors	Immunohistological and polymerase chain reaction (PCR) analyses, routine histology and immunocytochemistry analyses, repair tissue grading	In vivo	Positive collagen type II and aggrecan labeling, repair scores for chondroprogenitors ranged from 7 (abnormal) to 10 (nearly normal)	Formed a cartilage-like repair tissue	2010	Williams et al. [6]
	Chondroprogenitor cells	Hyaline cartilage	Sheep	Growth plate defects at the margin of the medial aspect of the proximal tibiae	A graft: endochondrally ossifiying cartilage from the peripheral margin of the secondary center of ossification and the adjacent zone of Ranvier tissue	Radiological assessment of longitudinal growth, histological analysis	In vivo	Endochondral ossification continued and no shortening no deformity resulted	Survived and persisted as cartilaginous tissue but was unable to restore, repair or function as a growth plate	1994	Wirth et al. [45]
	Chondroprogenitor cells	Articular cartilage	Bovine	Thigh muscle of severe-combined immunodeficient (SCID) mice	Intramuscular injection	Cryosectioning and PCR analyses	In vivo	Expressed sox9 and type II collagen	Survived but failed to create a robust cartilage pellet	2014	Marcus et al. [46]
Meniscus	Cartilage-derived progenitor cells (C-PCs)	Knee articular cartilage	Human	A radial tear in the inner anterior horn of the rat meniscus	An explant organ culture	Tissue immunohistochemistry and staining, messenger RNA expression, cell surface marker, stem cell differentiation and western blot analyses	In vitro	Elevated sox9 expression, maintained lower expression of type X collagen, resisted cellular hypertrophy and terminal differentiation, mobilized in response to chemokine signaling SDF-1/CXCR4 axis	Had the reparative ability to bridge and reintegrate torn meniscal fibrocartilage	2019	Jayasuriya et al. [47]
	Meniscus-derived mesenchymal stem cells (MMSCs)	Medial and lateral menisci	Rabbit	A wound with 1 mm diameter in the center of each meniscus	An explant organ culture and a graft: the Matrigel with cells used for implantation into nude rat skin	Histochemistry, immunocytochemistry, real-time quantitative polymerase chain reaction (RT-qPCR) and western blotting analyses	In vivo and in vitro	A pronounced tendency to chondrogenic differentiation, homing traits, more formation of cartilage-related proteins	Served as an alternative cell therapy in repairing damaged meniscus	2015	Ding and Huang [19]
	Human meniscus stem/progenitor cells (hMeSPCs)	Meniscus	Human	The removal of the anterior half of the rat medial meniscus and an experimental OA model	Intra-articular injection	Evaluation of multipotent differentiation potential, colony formation assay and expression analysis of meniscus-related genes, chemotaxis assay, cell labeling and detection, histology, transmission electron microscopy and immunostaining analyses	In vivo	More neo-tissue formation and better-defined shape but also resulted in more rounded cells and matured extracellular matrix, reduced expression of OA markers such as collagen I, collagen X, and hypoxia-inducible factor 2a (HIF-2a) but increased expression of collagen II	Enhanced the regeneration of injured meniscus through induced cell homing via the SDF-1/CXCR4 chemokine axis	2014	Shen et al. [18]
	Allogenous meniscus-derived stem cells (MeSCs)	Meniscus	Rabbit	The removal of the anterior half of the rabbit medial meniscus and a rabbit early experimental OA model	Intra-articular injection	Cell labeling and detection, radiographic evaluation, histology, immunohistochemistry, transmission electron microscopy, real-time PCR, biomechanical evaluation	In vivo	Did not elicit immunological rejection, but promoted neo-tissue formation with better-defined shape and more matured extracellular matrix, further protected joint surface cartilage and maintained joint space	Evoked a new strategy for articular cartilage protection and meniscus regeneration	2013	Shen et al. [17]
	Chondrocyte-derived progenitor cells (CDPCs)	Knee articular cartilage	Human	Large knee cartilage defects (6–13 cm^2^) in 15 patients	A graft: a collagen type I/III scaffold seeded with the CDPCs	Clinical evaluation, MRI evaluation, histology, pain and functional evaluation	In vivo	Significantly improved IKDC and Lysholm scores, the presence of chondrocyte-like cells and hyaline cartilage-like structure and matrix, reduced knee pain and swelling, and disappeared locking sensation	Supported the possibility of using in vitro the amplified CDPCs, for joint repair	2016	Jiang et al. [48]
TMJ condyle	Fibrocartilage stem cells (FCSCs)	TMJ condyle	Rat	A 2.5 mm perforation in the rabbit TMJ disc bilaterally and secondary OA	Intra-articular injection of the Wnt inhibitor sclerostin (SOST)	Histology, histomorphometry, and fluorescence-activated cell sorting (FACS) analyses	In vivo	Sustained the FCSC pool, improved TMJ gross morphology and proteoglycan distribution, reduced joint swelling	Exploited endogenous FCSCs to regenerate and repair cartilage	2016	Embree et al. [22]

When hyaline cartilage-derived pluripotent cells are transplanted into artificially constructed cartilage defects of the femur, high expression of type II collagen, and cartilage-like repair tissue formation were observed, and these results have been successfully verified in equine [44] and goat models [6]. However, this conclusion may not hold in some cases [45, 46]. Marcus et al. [46] demonstrated that when bovine articular cartilage-derived pluripotent cells were intramuscularly injected into a severe-combined immunodeficient (SCID) mouse, these cells could survive within the muscle mass but failed to produce cartilage-like tissue despite expressing Sox9 and type II collagen. These findings suggest that the cells may require further signals and a more favorable environment for chondrogenic differentiation.

In culture of man-made injured meniscus explants in vitro, meniscus-derived pluripotent cells could bridge and reintegrate torn meniscal fibrocartilage along the tear channel, as evidenced by the migratory ability in response to the chemokine signaling stromal-derived factor-1/stromal-derived factor-1 receptor (SDF-1/CXCR4) axis, a pronounced tendency toward chondrogenic differentiation, a greater than 100% increase in fibrochondrocyte proliferation, the elevated expression of Sox9 and decreased expression of type X collagen, and the resistance to cellular hypertrophy and terminal differentiation during the tissue repair process in a rat [47] and a rabbit [19] model. On this basis, Jayasuriya et al. [47] proposed that the initiation of the observed meniscal tissue repair is possible without first forming a blood clot, provided that an influx of stem cells is readily available near the damage site. Furthermore, the intra-articular injection of meniscus-derived pluripotent cells can enhance the regeneration of the injured meniscus at an early stage of OA, promoting neotissue formation with an improved shape and increased mature ECM and resulting in reduced expression of OA markers such as type I collagen, type X collagen, and hypoxia-inducible factor 2a (HIF-2a) but increased expression of collagen II [17, 18]. Notably, Jiang et al. [48] discovered a class of human chondrocyte-derived progenitor cells (CDPCs) and transplanted them into patients with large knee cartilage defects, leading to reduced knee pain and swelling and eliminating the locking sensation, which supported the possibility of cartilage-derived pluripotent cell-based therapies for human joint cartilage repair.

In addition to transplantation and intra-articular injection of cartilage-derived pluripotent cells, endogenous CPCs can be exploited to initiate cartilage regeneration and repair with drugs. Embree et al. [22] demonstrated the therapeutic application of sclerostin (SOST) in a rabbit model of injury to the TMJ disc and secondary OA; this molecule is an exogeneous canonical Wnt inhibitor, as canonical Wnt signals may be enhanced in diseased human TMJ condylar fibrocartilage.

However, there are some drawbacks of the current studies on cartilage-derived pluripotent cell-based therapies for joint cartilage repair. One is the use of stable cell lines because these cell lines may deviate from the primary cells that were used to generate them in vitro. The other is that animal cartilage is different from human cartilage in terms of its main structural features, including cellular distribution, vascularity, and collagen structure. Therefore, future research should focus on the application of cartilage-derived pluripotent cells in the treatment of human joint cartilage diseases.

## Conclusions

Stem cell-based therapy is a promising approach for joint cartilage repair. Currently used cell sources include autologous chondrocytes and MSCs, while in situ cartilage-derived pluripotent cell populations, present at target sites for cartilage repair, have become a research hotspot in recent years to determine whether they show improved repair of cartilage injury. These studies have determined and analyzed the cytological features and functions of cartilage-derived pluripotent cells, including increased chondrogenic protein expression during cartilage development, surface markers, colony formation, pluripotent differentiation, and migratory ability, to provide evidence for their ability to repair cartilage injury compared to that of autologous chondrocytes or MSCs. Although many studies have demonstrated the joint cartilage reparative capability of in situ cartilage-derived pluripotent cells residing in hyaline cartilage, meniscus, and TMJ condyle, evidence from clinical trials is lacking. Hence, the effectiveness and mechanisms of cartilage-derived pluripotent cell-based therapies for human joint cartilage repair remain to be further elucidated.

## Data Availability

The datasets generated and analyzed during the current study are available in the PubMed repository, www.ncbi.nlm.nih.gov/pubmed.

## Abbreviations

BMP-7: Bone morphogenic protein-7
BMSCs: Bone marrow-derived stem cells
CC: Condylar cartilage
CCs: Cartilage cells
CDPCs: Chondrocyte-derived progenitor cells
CFE: Colony-forming efficiency
COMP: Cartilage oligomeric protein
CPCs: Chondrogenic progenitor cells/Chondroprogenitor cells
ECM: Extracellular matrix
ES: Early senescent
FCSCs: Fibrocartilage stem cells
HCCs: High-efficiency colony-forming cells
HIF-2a: Hypoxia-inducible factor-2a
hMeSPCs: Human meniscus stem/progenitor cells
LS: Late senescent
MCP: Metacarpophalangeal
MeSCs: Meniscus-derived stem cells
MSCs: Mesenchymal stem cells
OA: Osteoarthritis
PD: Population doublings
SCID: Severe-combined immunodeficient
SDF-1: Stromal derived factor-1
SOST: Sclerostin
SZ: Superficial zone
SZP: Superficial zone protein
TMJ: Temporomandibular joint
